# Surveillance of Endoscopes: Comparison of Different Sampling Techniques

**DOI:** 10.1017/ice.2017.115

**Published:** 2017-06-21

**Authors:** Lien Cattoir, Thomas Vanzieleghem, Lisa Florin, Tania Helleputte, Martine De Vos, Bruno Verhasselt, Jerina Boelens, Isabel Leroux-Roels

**Affiliations:** 1 Department of Laboratory Medicine, Ghent University Hospital, Ghent, Belgium; 2 OneLIFE, Louvain-la-Neuve, Belgium; 3 Endoscopy Unit, Department of Gastroenterology, Ghent University Hospital, Ghent, Belgium; 4 Department of Clinical Chemistry, Microbiology, and Immunology, Ghent University, Ghent, Belgium; 5 Infection Control Team, Ghent University Hospital, Ghent, Belgium

## Abstract

**OBJECTIVE:**

To compare different techniques of endoscope sampling to assess residual bacterial contamination.

**DESIGN:**

Diagnostic study.

**SETTING:**

The endoscopy unit of an 1,100-bed university hospital performing ~13,000 endoscopic procedures annually.

**METHODS:**

In total, 4 sampling techniques, combining flushing fluid with or without a commercial endoscope brush, were compared in an endoscope model. Based on these results, sterile physiological saline flushing with or without PULL THRU brush was selected for evaluation on 40 flexible endoscopes by adenosine triphosphate (ATP) measurement and bacterial culture. Acceptance criteria from the French National guideline (<25 colony-forming units [CFU] per endoscope and absence of indicator microorganisms) were used as part of the evaluation.

**RESULTS:**

On biofilm-coated PTFE tubes, physiological saline in combination with a PULL THRU brush generated higher mean ATP values (2,579 relative light units [RLU]) compared with saline alone (1,436 RLU; *P*=.047). In the endoscope samples, culture yield using saline plus the PULL THRU (mean, 43 CFU; range, 1–400 CFU) was significantly higher than that of saline alone (mean, 17 CFU; range, 0–500 CFU; *P*<.001). In samples obtained using the saline+PULL THRU brush method, ATP values of samples classified as unacceptable were significantly higher than those of samples classified as acceptable (*P*=.001).

**CONCLUSION:**

Physiological saline flushing combined with PULL THRU brush to sample endoscopes generated higher ATP values and increased the yield of microbial surveillance culture. Consequently, the acceptance rate of endoscopes based on a defined CFU limit was significantly lower when the saline+PULL THRU method was used instead of saline alone.

*Infect Control Hosp Epidemiol* 2017;38:1062–1069

Flexible endoscopes are frequently used for diagnostic and therapeutic interventions. They are semicritical devices because they encounter mucous membranes and are reprocessed using high-level disinfection destroying all microorganisms except small numbers of bacterial spores.^1^ Due to their complex design with several narrow and long lumens, flexible endoscopes are difficult to clean and disinfect. The estimated incidence of infections associated with gastrointestinal endoscopy is low (1 in 1.8 million procedures).^1^ ^,^ ^2^ Nevertheless, contaminated endoscopes are among the medical devices most frequently linked to healthcare-associated outbreaks.^3^ Moreover, because most reported outbreaks involve multidrug-resistant organisms, it is likely that most outbreaks are being missed.^4^

Pathogen transmission is most often related to failure to comply with established cleaning and disinfection guidelines or with the use of defective equipment.^1^ Manual cleaning and drying are critical steps in reprocessing flexible endoscopes. Manual cleaning reduces the initial bioburden, enabling high-level disinfection to adequately decontaminate the endoscopes.^1^ Endoscope drying reduces the risk of bacterial proliferation during endoscope storage.^5^ ^,^ ^6^ Another potential risk is biofilm growth inside endoscope channels,^7^ ^,^ ^8^ which compromises disinfection and facilitates microbial transmission.^1^ ^,^ ^6^ ^–^ ^8^

Possibly, early detection of endoscope contamination using microbiological surveillance could prevent cross-transmission and infection of patients.^1^ ^,^ ^6^ Most European guidelines recommend routine surveillance of flexible endoscopes using the culture method. In the United States, there are currently no guidelines for routine monitoring,^9^ and agreement is lacking among guidelines regarding acceptance criteria, testing frequency, sampling technique, culture medium, and incubation conditions (Table 1).^5^

**TABLE 1:** Overview of Guidelines on Microbial Surveillance of Endoscopes

Guideline	Year	Frequency of Routine Samples	Sampling Technique	Sampling Volume, mL	Volume Used for Culture	Culture Medium	Incubation Temperature, °C	Duration of Incubation, d	Criterion of Acceptance
SHC, Belgium^10^	2010	Annually	Flushing with sterile saline	20 per channel	20 mL	…	…	…	Unclear
SFERD, Netherlands^11^	2014	None	Flushing with sterile saline +brush	20 per channel	20 mL	…	…	…	<20 CFU/channel
CTINILS, France^12^	2007	Annually	Flushing with sterile tensioactive fluid	100–200	100–200 mL	Non-selective agar	30	5	<25 CFU; no indicator MO
BSG, United Kingdom^13^	2008	None	…	…	…	…	…	…	…
ESGE-ESGENA, Europe^14^	2008	4x/year; annually	Flushing with sterile saline	20 per channel	1 mL	Non-selective agar	30	2	<20 CFU/mL; no indicator MO
GESA-GENCA, Australia^15^	2010	Depending on the type of scope	Flushing with sterile water or saline + brush	10 per channel	100 µL (after centrifugation)	2 blood agars	28 35	7	<10 CFU; no indicator MO
MACID, Canada^16^	2000	None or 2; 3×/year	Flushing with sterile water + brush	10	100 µL	Blood agar, Sabouraud agar	37 30	2 5	<20 CFU/0.1 mL
ASGE-SHEA, United States^17^	2011	None	…	…	…	…	…	…	…
APIC, United States^18^	2000	None	Flushing with sterile saline + brush	…	…	…	…	…	No vegetative bacteria

NOTE. MO, microorganisms; CFU, colony-forming units; … , not mentioned.

Because the sensitivity of different sampling strategies may vary, we aimed to compare different techniques of sampling flexible endoscopes. We compared 4 techniques reflecting current guidelines: flushing with sterile physiological saline (PHYS), flushing with neutralizing pharmacopeia diluent (NPD), and 2 flush-brush-flush techniques using PHYS in combination with the Olympus single-use, dual-ended cleaning brush or the PULL THRU brush.

## METHODS

### Endoscope Model

#### Endoscope model and sampling

For the endoscope model, we used polytetrafluoroethylene (PTFE) tubes with a 2.4-mm internal diameter and a 20-cm length (volume, 0.91 mL). Next, 20 PTFE tubes were each flushed with 1 mL from a positive hemoculture containing *Klebsiella pneumoniae* or *Escherichia coli* and were kept at room temperature for 24 hours (ie, non–biofilm-coated PTFE tubes). In addition, 2×20 PTFE tubes coated with biofilm (2 batches) were produced according to ISO 15883-5 Annex F and HTM 2030 standards that describe a model for growing biofilms representative of contamination inside an endoscope channel (ie, biofilm-coated PTFE tubes). Compared to the ISO standard, thinner PTFE tubes, closer to the actual size of endoscope channels, were used. Moreover, in addition to *Pseudomonas aeruginosa* (CIP A22), 2 relevant bacterial species (*Klebsiella pneumoniae* ATCC600703 and *Staphylococcus epidermidis* ATCC35984) were added to the biofilm to increase robustness.

We performed 4 sampling techniques 5 times on these PTFE tubes: (1) flushing with 10 mL PHYS (ie, 10PHYS), (2) flushing with 10 mL NPD (ie, 10NPD), (3) flush-brush-flush using 10 mL PHYS and a standard cleaning brush (Olympus, Hamburg, Germany) (ie, 10PHYS+SB), or (4) a PULL THRU brush (Medivators, Minneapolis, MN) (ie, 10PHYS+PT). Fluids (and brush tips) were collected in sterile containers. Moreover, 2 non–biofilm-coated PTFE tubes and 1 biofilm-coated PTFE tube were used as positive controls; they were cut into small pieces that were collected in sterile containers filled with 10 mL reverse osmosis water. These containers were then vortexed for 30 seconds, sonicated for 5 minutes, and vortexed again for 30 seconds. All samples and positive controls were processed for adenosine triphosphate (ATP) measurement and culture at the microbiology laboratory within 1 hour.

#### ATP measurement and microbial culture

ATP measurement was performed in duplicate using the Aquasnap Total test (Hygiena, Watford, UK) according to the manufacturer’s instructions with the SystemSURE Plus luminometer, except for NPD (due to interference with ATP quantification). Additionally, samples were diluted (1:10,000), and 100 µL was plated on trypticase soy agar (TSA), which was incubated for 7 days at 30°C. The total number of colony-forming units (CFU) was recorded.

### Endoscopes

Ghent University Hospital hosts 42 endoscopes and 5 automated endoscope reprocessors (AER; ETD3, Olympus, Hamburg, Germany). The reprocessing cycle consists of (1) bedside precleaning, (2) manual leak testing, (3) cleaning in the cleaning facility using the Olympus standard cleaning brush, (4) mechanical leak testing, and (5) high-level disinfection using glutaraldehyde in the AER. With the exception of gastroscopes, which are stored in storage cabinets, all flexible endoscopes are stored in endoscope drying cabinets.

#### Samples

After distinct reprocessing procedures, we compared 2 sampling techniques on a subset of 40 endoscopes each: 10 gastroscopes, 10 coloscopes, 5 endoscopic retrograde cholangiopancreatography (ERCP) scopes, 5 echo-endoscopes, and 10 bronchoscopes. The commissioning date of each endoscope was recorded. Flushing of 100 mL PHYS (ie, 100PHYS) was performed because most guidelines recommend this technique. A 10-fold higher volume was used than with the endoscope model because of the greater length of the endoscope tubes.

A flush-brush-flush technique using PHYS and PULL THRU brush (ie, 100PHYS+PT) was selected as second technique based on the endoscope model results. The 100PHYS+PT method consists of flushing endoscope channels with 50 mL PHYS, brushing the biopsy channel using a PULL THRU brush, and flushing again with 50 mL PHYS. In addition, a special brush (MyBrush, Olympus, Hamburg, Germany) was used to sample the forceps elevator recess of ERCP scopes. Fluid was collected in sterile containers with the brush tip(s). To include all channels, a sterile connector (MAJ-621, Olympus, Hamburg, Germany) was used to flush the endoscopes (except for bronchoscopes having only 1 channel). Because ATP tests and PULL THRU brushes are not sterile, they were cultured 10-fold as a negative control.

#### ATP measurement and microbial culture

An Aquasnap Total test was performed on all samples. The remaining sample was filtered through a 0.45-µm membrane using an EZ-Stream pump (Merck Millipore, Molsheim, France). This membrane was put on TSA agar, which was incubated for 7 days at 30°C. The CFU count was recorded daily (except weekends), and matrix-assisted laser desorption/ionization time-of-flight mass spectrometry (MALDI-TOF MS; Microflex LT, Bruker Daltonics, Bremen, Germany) was used to identify indicator microorganisms: Enterobacteriaceae, *Pseudomonas aeruginosa* and other *Pseudomonas* spp., *Stenotrophomonas malthophilia*, *Acinetobacter* spp., *Staphylococcus aureus*, and *Candida* spp.^5^ ^,^ ^12^

### Statistical Analysis

Culture results obtained from non–biofilm- and biofilm-coated PTFE tubes were expressed as recovery rate compared to positive controls. Mean and 95% confidence intervals (CI) of ATP and culture results were calculated for each sampling technique. Results were displayed in box-and-whisker plots. Statistical differences between sampling techniques were evaluated using the Kruskall-Wallis test (>2 groups) and the Mann-Whitney test (2 groups). *P*<.05 was considered statistically significant. Data from the endoscopes were also compared with French National Technical Committee on Nosocomial Infection (FNTCNI) criteria: (1) <25 CFU per endoscope and (2) absence of indicator microorganisms.^12^ The χ^2^ test was used to compare the proportion of unacceptable samples. *P*<.05 was considered statistically significant.

A scatterplot and the Spearman correlation coefficient were used to check for a linear relationship between ATP and culture results. *P*<.01 was considered statistically significant. ATP values of endoscope samples with acceptable versus unacceptable culture results (based on FNTCI criteria) were compared, both for the entire group and for the 100PHYS and 100PHYS+PT subgroups. Receiver operator curve (ROC) analysis was used to determine the optimal cutoff ATP value. All statistical analyses were conducted using SPSS version 23 statistical software (IBM, Armonk, NY).

## RESULTS

### Endoscope Model

Mean ATP values obtained using different sampling techniques on non–biofilm-coated PTFE tubes were comparable: 5,574 for 10PHYS, 4,454 for 10PHYS+SB, and 5,014 for 10PHYS+PT (*P*=.37). Conversely, differences in ATP results using biofilm-coated tubes were significant (*P*=.045) (Table 2). In a pairwise comparison, only the difference between the 10PHYS and 10PHYS+PT subgroups was retained as statistically significant (*P*=.047).

**TABLE 2:** Mean ATP Results and Culture Yield of Different Sampling Techniques Performed on an Endoscope Model

	Mean ATP Value (95% CI)		Mean Yield of Culture, % (Recovery Rate^a^) (95% CI)	
Sampling technique	Non–biofilm PTFE	Biofilm PTFE	Non–biofilm PTFE	Biofilm PTFE
10PHYS	5,574 RLU (4,713–6,434)	1,436 RLU (901–1,970)^b^	34 (11–57)	59 (47–71)
10NPD	…	…	32 (12–52)	44 (25–63)
10PHYS+SB	4,454 RLU (3,297–5,610)	1,408 RLU (915–1,901)	22 (6–37)	37 (13–60)
10PHYS+PT	5,014 RLU (4,104–5,924)	2,579 RLU (1,623–3,536)^b^	16 (5–26)	57 (35–79)

NOTE. 10PHYS, flushing with 10 mL sterile physiological saline; 10NPD, flushing with 10 mL NPD; 10PHYS+SB, flush-brush-flush using 10 mL sterile physiological saline and a standard cleaning brush; 10PHYS+PT, flush-brush-flush using 10 mL sterile physiological saline and a PULL THRU brush; ATP, adenosine triphosphate; RLUs, relative light units; CI, confidence interval; CFU, colony-forming units; PTFE, polytetrafluoroethylene; …, experiment not performed because of interference of yellow-colored NPD solutions with measurement of ATP.

a Percentage recovery of a certain technique compared to the positive controls.

b Statistically significant difference between mean ATP value of 10PHYS and 10PHYS+PT sampling methods on biofilm-coated PTFE tubes (*P*=.047).

Culture results are presented as percentage recovery compared to the positive control (Table 2). The mean number of CFUs using the 4 different sampling techniques did not differ statistically in either non–biofilm- or biofilm-coated tubes (*P*=.53 and *P*=.27, respectively). However, the 10PHYS and 10PHYS+PT techniques had the highest mean yields for biofilm-coated PTFE tubes, while the 10PHYS and 10NPD methods produced the highest mean yields for non–biofilm-coated tubes.

There was no correlation between ATP measurements and culture results (r_S_=−0.08; *P*=.56).

### Endoscopes

ATP and culture results varied widely with only a weak correlation (r_S_=0.38; *P*=.001). However, ATP values of samples classified as unacceptable based on FNTCNI criteria were higher compared to those classified as acceptable (*P*=.002). Subgroup analyses revealed that this finding was true only for samples obtained with the PHYS+PULL THRU brush method (*P*=.001) and not for the PHYS method alone (*P*=.9). An ATP cutoff value of >2 RLU on 100PHYS+PT samples was predictive for classification as unacceptable, with sensitivity and specificity of 87.5% and 71%, respectively.

**FIGURE 1:**
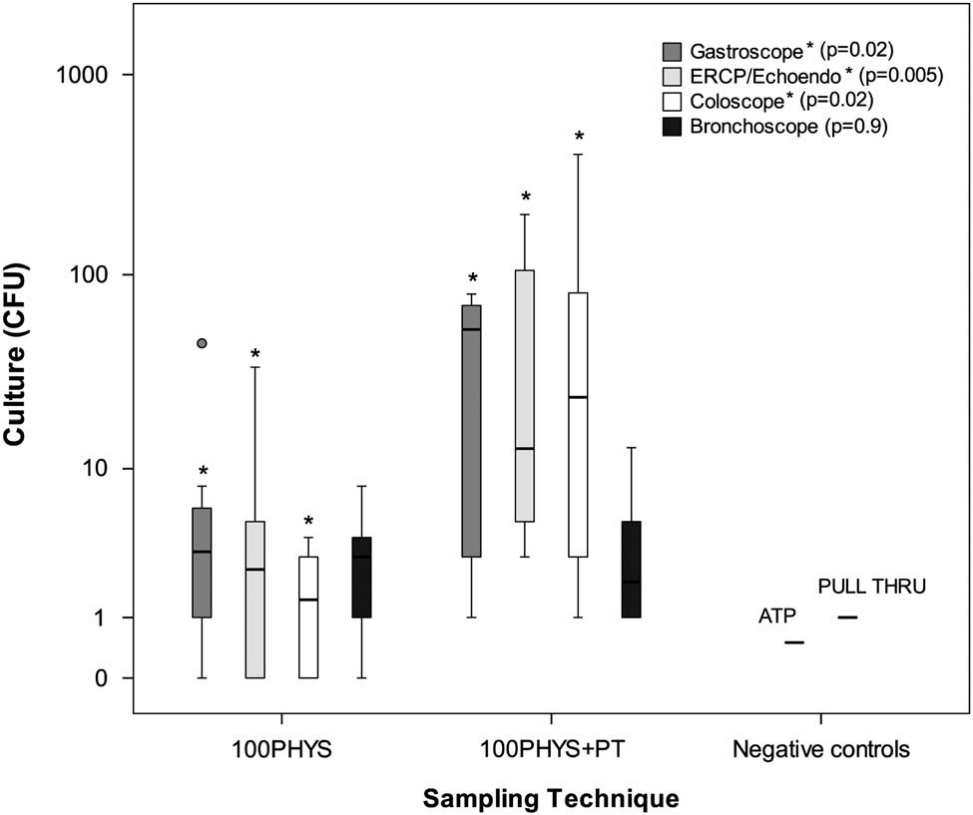
Culture results obtained from endoscopic samples using the 100PHYS and 100PHYS+PT sampling methods and the results of negative controls. Culture results were obtained from TSA agars with filter. Note: 100PHYS, flushing with 100 mL sterile physiological saline; 100PHYS+PT, flush-brush-flush using 100 mL sterile physiological saline and a PULL THRU brush; CFU, colony-forming units; ATP, adenosine triphosphate; *Statistically significant differences between mean yield of culture using 100PHYS and 100PHYS+PT sampling methods for different types of endoscopes. *P* values are shown.

Culture results showed important differences (Table 3). The culture yield using 100PHYS+PT sampling (mean, 43 CFU; range, 1–400 CFU) was significantly higher than for 100PHYS sampling (mean, 17 CFU; range, 0–500 CFU; *P*<.001). Subgroup analysis showed that addition of a PULL THRU brush to the sampling procedure resulted in higher culture results for all endoscope types, except for bronchoscopes. The CFU counts of negative controls for ATP tests (mean, 0.5 CFU; 95% CI, 0.1–0.9 CFU) and PULL THRU brushes (mean, 0.9 CFU;95% CI, 0.4–1.8 CFU) were negligible (Figure 1).

**TABLE 3:** ATP, Culture Results,^a^ and Acceptance Rates Obtained From Endoscope Samples Using 2 Different Sampling Techniques

		No. of Endoscopes	ATP (RLU) (95% CI)	Culture (CFU) (95% CI)	Indicator MO	Acceptance Rate
100PHYS	Gastroscopes	10	13 (2–23)	8 (0–17)	1/10	8/10
	ERCP/Echo-endoscopes	10	17 (1–33)	6 (0–13)	1/10	8/10
	Coloscopes	10	3 (0–8)	51 (0–164)	0/10	9/10
	Bronchoscopes	10	1 (0–1)	3 (1–5)	1/10^b^	8/10
	All endoscopes	40	8 (4–13)	17 (0–42)^c^	3/40 (7.5%)	33/40 (82.5%)^d^
100PHYS+PT	Gastroscopes	10	36 (14–58)	42 (18–69)	0/10	4/10
	ERCP/Echo-endoscopes	10	36 (0–76)	53 (5–100)	1/10	5/10
	Coloscopes	10	7 (0–19)	72 (0–158)	1/10	5/10
	Bronchoscopes	10	1 (0–1)	4 (1–6)	0/10	10/10
	All endoscopes	40	20 (8–31)	43 (19–66)^c^	2/40 (5%)	24/40 (60%)^d^

NOTE. 100PHYS, flushing with 100 mL sterile physiological saline; 100PHYS+PT, flush-brush-flush using 100 mL sterile physiological saline and a PULL THRU brush; RLUs relative light units; CFU, colony-forming units; MO, microorganisms; CI, confidence interval.

a Culture results were obtained from TSA agars with filter.

b In 1/10 bronchoscopes *Aspergillus fumigatus* was found.

c Statistically significant difference between mean culture yield using 100PHYS and 100PHYS+PT sampling (*P*<.001).

d Statistically significant difference between the number of samples classified as (un)acceptable using 100PHYS vs 100PHYS+PT (*P*=.03).

Indicator microorganisms were detected in 5 samples from different endoscopes: 2 *Pseudomonas* species, 2 *Pseudomonas putida*, and 1 *Acinetobacter iwoffii*. Overall, 3 indicator microorganisms were obtained from 100PHYS samples, and 2 were obtained from 100PHYS+PT samples. In a single bronchoscope sample obtained using 100PHYS+PT, *Aspergillus fumigatus* was detected. Results (ie, total CFU, ATP and identified microorganisms) of all endoscope samples are shown in Online Supplementary Table 1. Identified microorganisms were mainly skin commensals (eg, coagulase negative staphylococci and *Micrococcus luteus*) and environmental bacteria (eg, *Bacillus* spp.).

Using French acceptance criteria, the number of samples classified as unacceptable was significantly higher using the 100PHYS+PT technique (ie, 16 of 40) compared with the 100PHYS only method (ie, 7 of 40; *P*=.03). The age of the endoscopes was comparable between both groups (ie, 4.6 years for 100PHYS vs 4.8 years for 100PHYS+PT); no correlation was detected between endoscope age and culture results (r_s_=−0.07; *P*=.6).

In 16 of 80 samples, CFU counts were not recorded at 48 hours because plates were not read during weekends. From the remaining 64 samples, only 4 (6%) developed growth after 48 hours with 1–5 CFU and no indicator microorganisms. The other 60 (94%) samples either showed no growth (10 samples) or growth already developed at 48 hours (50 samples). In 35 of those 50 samples, there was no additional growth after 48 hours. The remaining 15 samples showed minor increases in CFU counts after 48 hours, but all indicator organisms grew within this time frame. Overall, all samples were classified correctly as (un)acceptable at 48 hours of incubation.

## DISCUSSION

Microbiological surveillance of endoscopes is influenced by culture method and sampling technique, especially its recovery rate. However, guidelines show major differences with respect to recommended technique. To discriminate performance of sampling techniques, 4 were selected: (1) flushing with PHYS, (2) flushing with NPD, (3) flush-brush-flush using PHYS and standard cleaning brush, and (4) flush-brush-flush using the PHYS+ PULL THRU brush method. Retrograde sampling (from distal to proximal end)^4^ was not included because it is not recommended in guidelines and is impractical. Samples were cultured on TSA agar after filtration. We processed our data using French acceptance criteria, which appear to have been based on expert opinion rather than on clinical evidence.^12^

ATP measurement was performed in addition to culture. It is quick (<1 minute) and simple, but it fails to detect small quantities of microorganisms,^21^ so it could be considered an indicator of endoscope cleanliness, notably to audit manual cleaning adequacy.^19^ ^,^ ^20^ Recommended maximum RLU values for samples taken at the end of reprocessing (during storage or just before reuse) are not available.

In vitro experiments revealed that, for biofilm-coated PTFE tubes, ATP values of 10PHYS+PT samples were significantly higher than those of 10PHYS samples. ATP values of non–biofilm-coated tubes were comparable among the 4 sampling techniques. Culture results showed that mean yield from biofilm-coated PTFE tubes was highest for 10PHYS and 10PHYS+PT techniques, whereas for non–biofilm-coated tubes 10PHYS and 10NPD produced the highest mean yield. However, differences in mean CFU count did not reach statistical significance. Taken together, because biofilm-coated PTFE tubes likely resemble the real-life situation more closely than non–biofilm-coated tubes, the PHYS+PULL THRU brush method was selected for comparison with PHYS alone, which is recommended for use on endoscopes by most guidelines because it is inexpensive and simple.

In our study, there was no correlation between ATP and culture results in in vitro experiments or in endoscope samples. This result corresponds to the findings of Batailler et al,^21^ who concluded that ATP cannot be used as an alternative to microbiological tests for monitoring endoscope reprocessing. However, according to our data, ATP seems to be able to distinguish samples classified as acceptable from samples classified as unacceptable. Subgroup analysis showed that this is only true for 100PHYS+PT samples, not for 100PHYS samples. Using an ATP cutoff value of >2 RLU for 100PHYS+PT samples, sensitivity and specificity were 87.5% and 71%, respectively. Applying this cutoff to our results, 31 of 40 samples would have been immediately classified correctly: 17 acceptable and 14 unacceptable. There were 7 false-positive results and 2 false-negative results; both had >25 CFU per endoscope, and 1 sample also grew indicator microorganisms. Due to the intrinsic inability of ATP to detect small numbers of microorganisms and based on our limited data, microbiological culture remains necessary and should not be omitted. The value of ATP in this setting and the ATP threshold to discriminate acceptable from unacceptable endoscopes needs to be validated in larger studies.

On endoscopes, the 100PHYS+PT method yielded significantly higher culture results than the 100PHYS only method. Mechanical action seems to facilitate the release of organic matter and microorganisms. Also, the number of endoscope samples classified as unacceptable using French acceptance criteria was significantly higher using the 100PHYS+PT method: 40% for 100PHYS+PT versus 17.5% for 100PHYS. Notably, these differences are not influenced by endoscope age. Analysis of negative controls shows that differences cannot be explained solely by the use of nonsterile brushes. Moreover, subgroup analysis revealed that adding a PULL THRU brush to the sampling procedure resulted in higher culture results for all types of endoscopes, except for bronchoscopes. The simpler design of bronchoscopes (1 channel only), compared to more complex gastrointestinal endoscopes, may account for this difference.

Physical removal of soil by complete surface contact between the circular rubber discs of the PULL THRU brush and the lumen wall probably explains the superiority of the PULL THRU brush over the standard cleaning brush. Based on our findings, it could be argued to replace standard cleaning brushes with PULL THRU brushes for manual endoscope cleaning. Because current evidence is limited, future research on the efficacies of different brush types for manual cleaning of flexible endoscopes is warranted.^22^

In our study, the final results were obtained at 48 hours of incubation because almost all positive endoscopes (50 of 54) developed growth within this time frame. These results contrast with other studies in which 30%–45% of endoscope samples became positive after >2 days of incubation.^5^ ^,^ ^23^ Different sampling and culture protocols impede direct comparison of results. In a study compiling the results of >1,000 samplings on gastrointestinal endoscopes, only 55.5% of all contaminated endoscopes were positive at 48 hours of incubation. The risk of contamination was significantly reduced when endoscopes were kept in storage cabinets (as in our setting).^5^ Despite the fact that culture methods used by Saliou et al are comparable with those used in our study, sampling methods and reprocessing methods were different. Notably, we did not use neutralizers, which are known to improve microbial recovery. Therefore, it is possible that slow-growing microorganisms, causing a change in endoscope classification after 2 days, were unable to survive in physiological saline between sampling and culture.^5^ Overall, the reduced incubation period of 48 hours might have an important impact on logistical issues and workload, but this aspect needs further validation prior to inclusion in a surveillance protocol.

To the best of our knowledge, only 1 other study compared efficacies of several sampling techniques for microbial surveillance of endoscopes. Aumeran et al^24^ used an experimental model of biofilm grown on endoscope internal tubing and performed an in-use evaluation sampling endoscopes during routine clinical practice with 2 different sampling solutions. They concluded that the use of tensioactive sampling fluid was significantly more efficient. However, brushing was not included in this study; thus, direct comparison of the results is difficult.

Our study has several limitations. Endoscopes were sampled after distinct reprocessing cycles. Although endoscope conditions differed between samplings, consecutive sampling on the same endoscope would induce a greater sampling bias. As mentioned above, we did not use any substance to neutralize remaining high-level disinfectant (glutaraldehyde in our case). It is also possible that other culture conditions, such as incubation temperature (eg, 35°C instead of 30°C) or different agar plates (eg, blood agar), would generate a higher yield or allow growth of different microorganisms. However, because the focus of this study was the evaluation of various sampling techniques, comparison of different culture methods could be the object of a separate study. Finally, our study was conducted in a single center on a limited number of endoscopes. It remains to be demonstrated whether our findings can be extrapolated to other settings, where, for example, peracetic acid instead of glutaraldehyde is being used.

In conclusion, sampling methods influence recovery rate and thus results and interpretation of microbial surveillance cultures of flexible endoscopes. The association of brushing using a PULL THRU brush to the endoscope sampling procedure increased the yield of microbial surveillance culture. However, generally accepted criteria for endoscope culture need to be defined, ideally based on clinical data regarding the risk of nosocomial transmission. Moreover, thresholds may need to be adjusted depending on the sensitivity of the sampling technique. The added value of ATP in the surveillance of endoscopes needs to be confirmed in future studies. In our study, all endoscopes were classified correctly as acceptable or unacceptable at 48 hours of incubation. However, given the concern about slow-growing microorganisms, it seems prudent to extend the incubation period to 7 days.

## Supplementary material

For supplementary material accompanying this paper visit http://dx.doi.org/10.1017/ice.2017.115. click here to view supplementary material
