# Supplementary material for: Surveillance of Endoscopes: Comparison of Different Sampling Techniques
Source: Infect Control Hosp Epidemiol. 2017 Jun 21;38(9):1062–9. doi: 10.1017/ice.2017.115 (PMC5647674; doi:10.1017/ice.2017.115)
Supplement: Supplementary file 1 [file S0899823X17001155sup001.docx]

|  | **ATP (RLU)** | **CFU** | **Identification of cultured microorganisms** |
| --- | --- | --- | --- |
| **100PHYS** |  |  |  |
| Gastroscope 1 | 29 | 8 | *Staphylococcus epidermidis, Bacillus species, Micrococcus luteus, Roseomonas mucosa* |
| Gastroscope 2 | 17 | 6 | *Bacillus species, Micrococcus luteus, Lysinibacillus species* |
| Gastroscope 3 | 3 | 5 | *Staphylococcus epidermidis, Staphylococcus haemolyticus, Micrococcus luteus, Moraxella osloensis* |
| Gastroscope 4 | 5 | 2 | *Bacillus cereus, unidentifiable micro-organism* |
| Gastroscope 5 | 3 | 2 | ***Pseudomonas species****, Paracoccus species* |
| Gastroscope 6 | 2 | 1 | *Bacillus species* |
| Gastroscope 7 | 11 | 0 |  |
| Gastroscope 8 | 4 | 45 | *Bacillus species, Brevibacterium species, Paracoccus yeei* |
| Gastroscope 9 | 47 | 6 | *Staphylococcus capitis, Staphylococcus epidermidis, Staphylococcus warneri* |
| Gastroscope 10 | 4 | 0 |  |
| Duodeno/Echo-endoscope 1 | 72 | 3 | *Bacillus species, Micrococcus luteus* |
| Duodeno/Echo-endoscope 2 | 4 | 0 |  |
| Duodeno/Echo-endoscope 3 | 1 | 0 |  |
| Duodeno/Echo-endoscope 4 | 5 | 2 | *Bacillus thermoamylovorans, unidentifiable micro-organism* |
| Duodeno/Echo-endoscope 5 | 7 | 5 | *Bacillus pumilus, Bacillus species, Paenibacillus species* |
| Duodeno/Echo-endoscope 6 | 20 | 34 | *Staphylococcus epidermidis, Micrococcus luteus, Corynebacterium propinquum, Kocuria species* |
| Duodeno/Echo-endoscope 7 | 28 | 5 | *Acremonium species* |
| Duodeno/Echo-endoscope 8 | 3 | 0 |  |
| Duodeno/Echo-endoscope 9 | 28 | 1 | *Moraxella osloensis* |
| Duodeno/Echo-endoscope 10 | 1 | 5 | ***Pseudomonas putida,*** *Micrococcus luteus* |
| Coloscope 1 | 21 | 4 | *Staphylococcus epidermidis, Staphylococcus warneri, Bacillus species, Brevibacillus parabrevis* |
| Coloscope 2 | 1 | 1 | *Kocuria palustris* |
| Coloscope 3 | 0 | 1 | *Bacillus species* |
| Coloscope 4 | 0 | 3 | *Staphylococcus epidermidis, Staphylococcus hominis* |
| Coloscope 5 | 0 | 0 |  |
| Coloscope 6 | 6 | 0 |  |
| Coloscope 7 | 2 | 2 | *Bacillus species, Micrococcus luteus* |
| Coloscope 8 | 0 | 0 |  |
| Coloscope 9 | 1 | 3 | *Staphylococcus epidermidis, Bacillus species* |
| Coloscope 10 | 1 | 500 | *Staphylococcus epidermidis, Staphylococcus warneri, Bacillus species, Brevibacterium species* |
| Bronchoscope 1 | 1 | 3 | *Staphylococcus epidermidis, Bacillus species, unidentifiable micro-organism* |
| Bronchoscope 2 | 0 | 3 | *Micrococcus luteus, unidentifiable micro-organism* |
| Bronchoscope 3 | 0 | 7 | ***Pseudomonas putida****, Moraxella osloensis, Micrococcus luteus, unidentifiable micro-organism* |
| Bronchoscope 4 | 0 | 0 |  |
| Bronchoscope 5 | 0 | 1 | *Micrococcus luteus* |
| Bronchoscope 6 | 0 | 3 | *Staphylococcus capitis, Staphylococcus epidermidis* |
| Bronchoscope 7 | 0 | 1 | *Unidentifiable micro-organism* |
| Bronchoscope 8 | 1 | 4 | *Bacillus cereus, Bacillus subtilis, Bacillus species, Roseomonas mucosa* |
| Bronchoscope 9 | 3 | 8 | *Bacillus species, Micrococcus luteus* |
| Bronchoscope 10 | 1 | 0 |  |
|  |  |  |  |
| **100 PHYS+PT** |  |  |  |
| Gastroscope 1 | 82 | 40 | *Staphylococcus epidermidis, Bacillus cereus, Brevibacillus parabrevis, Micrococcus luteus* |
| Gastroscope 2 | 70 | 75 | *Staphylococcus warneri, Bacillus flexus, Bacillus thuringiensis, Micrococcus luteus, Paracoccus yeei* |
| Gastroscope 3 | 15 | 80 | *Bacillus cereus, Brevibacillus parabrevis, Lysinibacillus sphaericus* |
| Gastroscope 4 | 8 | 70 | *Bacillus cereus, Brevibacillus parabrevis, Lysinibacillus sphaericus* |
| Gastroscope 5 | 63 | 70 | *Staphylococcus epidermidis, Bacillus species, Moraxella osloensis, unidentifiable micro-organism* |
| Gastroscope 6 | 11 | 3 | *Bacillus species, Micrococcus luteus, unidentifiable micro-organism* |
| Gastroscope 7 | 24 | 1 | *Staphylococcus hominis* |
| Gastroscope 8 | 10 | 13 | *Staphylococcus hominis, Bacillus cereus, Bacillus species, unidentifiable micro-organism* |
| Gastroscope 9 | 67 | 2 | *Staphylococcus saprophyticus, Roseomonas mucosa* |
| Gastroscope 10 | 8 | 70 | *Bacillus cereus, Bacillus megaterium, Bacillus species, Lysinibacillus sphaericus* |
| Duodeno/Echo-endoscope 1 | 2 | 10 | *Bacillus cereus, Paenibacillus species, Lysinibacillus sphaericus* |
| Duodeno/Echo-endoscope 2 | 2 | 15 | *Staphylococcus warneri, Bacillus cereus, Bacillus flexus, Brevibacillus parabrevis* |
| Duodeno/Echo-endoscope 3 | 21 | 5 | *Bacillus circulans, Micrococcus luteus, unidentifiable micro-organism* |
| Duodeno/Echo-endoscope 4 | 33 | 105 | *Bacillus cereus, Bacillus species, Lysinibacillus sphaericus, unidentifiable micro-organism* |
| Duodeno/Echo-endoscope 5 | 44 | 64 | *Bacillus species, Paenibacillus urinalis, Moraxella osloensis* |
| Duodeno/Echo-endoscope 6 | 32 | 11 | ***Acinetobacter Iwoffii****, Bacillus cereus* |
| Duodeno/Echo-endoscope 7 | 3 | 200 | *Staphylococcus capitis, Brevibacillus borstelensis* |
| Duodeno/Echo-endoscope 8 | 8 | 3 | *Brevibacillus species, Micrococcus luteus* |
| Duodeno/Echo-endoscope 9 | 22 | 3 | *Bacillus simplex, unidentifiable micro-organism* |
| Duodeno/Echo-endoscope 10 | 191 | 110 | *Bacillus weihenstephanensis, Brevibacterium parabrevis, Lysinibacillus sphaericus, Moraxella osloensis* |
| Coloscope 1 | 0 | 40 | ***Pseudomonas species****, Bacillus species, Moraxella osloensis* |
| Coloscope 2 | 2 | 100 | *Korucia palustris* |
| Coloscope 3 | 0 | 14 | *Streptococcus salivarius, Micrococcus luteus, unidentifiable micro-organism* |
| Coloscope 4 | 1 | 1 | *Moraxella osloensis* |
| Coloscope 5 | 0 | 6 | *Bacillus species, Moraxella osloensis, Roseomonas mucosa* |
| Coloscope 6 | 0 | 3 | *Bacillus flexus, Micrococcus luteus* |
| Coloscope 7 | 0 | 2 | *Staphylococcus hominis, unidentifiable micro-organism* |
| Coloscope 8 | 53 | 72 | *Bacillus idriensis, Bacillus weihenstephanensis, Bacillus species, Lysinibacillus fusiformis* |
| Coloscope 9 | 9 | 400 | *Bacillus species* |
| Coloscope 10 | 4 | 81 | *Staphylococcus epidermidis, Staphylococcus pasteuri, Bacillus species* |
| Bronchoscope 1 | 1 | 2 | *Brevibacillus parabrevis* |
| Bronchoscope 2 | 2 | 1 | *Brevibacillus parabrevis* |
| Bronchoscope 3 | 0 | 1 | *Staphylococcus epidermidis* |
| Bronchoscope 4 | 0 | 5 | *Bacillus cereus, Brevibacillus parabrevis, Micrococcus luteus* |
| Bronchoscope 5 | 0 | 1 | *Micrococcus luteus* |
| Bronchoscope 6 | 0 | 3 | *Bacillus cereus, Brevibacillus parabrevis, Brachybacterium species* |
| Bronchoscope 7 | 0 | 2 | *Paenibacillus species, Micrococcus luteus* |
| Bronchoscope 8 | 1 | 13 | *Bacillus melaninogenicus, Bacillus subtilis, Bacillus weihenstephanensis, Paenibacillus species, Micrococcus luteus, unidentifiable micro-organism* |
| Bronchoscope 9 | 1 | 7 | *Aspergillus fumigatus, Staphylococcus warneri, Bacillus species, Micrococcus luteus* |
| Bronchoscope 10 | 1 | 1 | *Bacillus circulans* |

Culture results are obtained from TSA agars with filter; 100PHYS, flushing with 100 mL of physiological saline; 100PHYS+PT, flush-brush-flush using 100 mL of physiological saline and a PULL THRU^TM^ brush; RLUs, Relative Light Units; CFU, Colony Forming Units.
Indicator microorganisms according to the French guideline are indicated in bold.^12^
